# Predicting New Materials for Hydrogen Storage Application

**DOI:** 10.3390/ma2042296

**Published:** 2009-12-14

**Authors:** Ponniah Vajeeston, Ponniah Ravindran, Helmer Fjellvåg

**Affiliations:** Center for Materials Sciences and Nanotechnology, Department of Chemistry, University of Oslo, Box 1033 Blindern N-0315, Oslo, Norway

**Keywords:** hydrogen storage materials, theoretical modeling, complex hydrides, structural study

## Abstract

Knowledge about the ground-state crystal structure is a prerequisite for the rational understanding of solid-state properties of new materials. To act as an efficient energy carrier, hydrogen should be absorbed and desorbed in materials easily and in high quantities. Owing to the complexity in structural arrangements and difficulties involved in establishing hydrogen positions by x-ray diffraction methods, the structural information of hydrides are very limited compared to other classes of materials (like oxides, intermetallics, *etc.*). This can be overcome by conducting computational simulations combined with selected experimental study which can save environment, money, and man power. The predicting capability of first-principles density functional theory (DFT) is already well recognized and in many cases structural and thermodynamic properties of single/multi component system are predicted. This review will focus on possible new classes of materials those have high hydrogen content, demonstrate the ability of DFT to predict crystal structure, and search for potential meta-stable phases. Stabilization of such meta-stable phases is also discussed.

## 1. Introduction

The decreasing fossil fuel supply and growing number of densely populated metropolitan cities with poor local air quality have spurred an initiative to develop an alternative fuel. Hydrogen, which may be produced from renewable sources while burning pollution-free, has emerged as one of the most promising candidates for the replacement of the current carbon-based energy systems. Interest in hydrogen as a fuel has grown dramatically since 1990, and many advances in hydrogen production and utilization technologies have been made. However, hydrogen storage technologies must be significantly advanced if a hydrogen-based energy system, particularly in the transportation sector, is to be established. Hydrogen can be made available on-board vehicles in containers of compressed or liquefied H${}_{2}$, in metal hydrides, via chemical storage or by gas-on-solid adsorption. Although each method possesses desirable characteristics, no approach satisfies all the requirements such as efficiency, size, weight, cost and safety for transportation or utility use. Gas-on-solid adsorption is an inherently safe and potentially high energy density hydrogen storage method that could be extremely energy efficient. Long-term system targets include reversible H${}_{2}$ discharge of >9 wt % and >81 kg H${}_{2}$/m${}^{3}$ at moderate pressures and temperatures with rapid charging and discharging kinetics, high H${}_{2}$ purity, acceptable cost, and long operational life. Presently, a compact, lightweight hydrogen-storage system for transportation applications with affordable temperature is not available. Hydrogen storage is therefore the key technology that must be significantly advanced in terms of performance and cost effectiveness, if hydrogen is to become an important part of the world’s energy economy.

Crystallization plays an important role in various industries as a large-scale technique for separation, purification, and structure determination. Most of the compounds crystallize at some point during their production process. Knowledge about the crystal structures is a prerequisite for the rational understanding of the solid-state properties of new materials. The current interest in the development of novel metal-hydrides stems from their potential use as reversible hydrogen storage devices at low and medium temperatures. The crystal structure, shape, size, and surface composition of materials are major factors that control the hydrogen sorption properties for energy storage applications. To act as an efficient energy carrier, hydrogen should be absorbed and desorbed in materials easily and in high quantities. Also, in order to use them in practical applications, the materials involved in such compounds should be easily available in large quantities with cheaper price. Alkali- and alkaline-earth-based complex hydrides are expected to have a potential as viable modes for storing hydrogen at moderate temperatures and pressures [1,2,3,4,5,6,7]. These hydrides (e.g., LiAlH${}_{4}$, NaAlH${}_{4}$, Li${}_{3}$BN${}_{2}$H${}_{8}$, *etc.* [1,2,3,4,5,6,7,8]) have higher hydrogen storage capacity at moderate temperatures than conventional hydride systems based on intermetallic compounds. The disadvantage for the use of these materials for practical applications is the lack of reversibility and poor kinetics. Recent experimental findings have shown that the decomposition temperature for certain complex hydrides can be modified by introduction of additives [3,4] and/or reduction of particle size [9,10,11,12]. This has opened up research activities on identification of appropriate admixtures for known or hitherto unexplored hydrides. To date, none meets Department of energy’s (USA) targets for storing and releasing enough hydrogen fuel on demand [13,14].

In this review we have covered the following topics. The prediction of complex hydrides crystal structures based on total-energy studies is in the second part. It is well known that most of the complex hydrides have well-defined chemical formula and are perfectly stoichiometric compounds (the hydrogen occupancy is always one). Hence, we considered only the stoichiometric defect-free compounds in this study. In the third part, we discussed the challenges and the limitation of such structural prediction from the density functional studies. In the fourth part we demonstrated how one can use DFT as a tool to identify potential meta-stable phases. Finally, how one can stabilize such predicted meta-stable phases by substitution is discussed in the fifth part.

## 2. Prediction of Hydride Crystal Structures

### 2.1. Structural complexity

The crystal structures of pure elements and most of the binary compounds have been frequently studied and are well characterized. On turning to ternary compounds, however, the amount of knowledge is considerably less extensive (an estimated 10% coverage of structural information) and for quaternary and multi-component phases the structural knowledge is extremely poor.

The knowledge on hydrides fits nicely into this general picture, but it must be recalled that structure determination of hydrides is confronted with an additional complexity that hydrogen is by far the lightest element. X-ray diffraction (XRD) techniques are suitable for determining the dimensions of the unit cell and the positions of the non-hydrogen atoms, but it is a poor technique to identify the hydrogen positions in hydrides. Neutron diffraction (ND) is also a poor tool to locate hydrogen in crystal lattices, however if one uses deuterated compounds it is very powerful. The problem to use deuterated sample is that the deuterium isotope is quite expensive and its scarcity has so far limited the application of this technique. Neutron diffraction is indeed suitable method for locating the position of both metal and deuterium atoms in deuterides. Neutrons are scattered by nuclei, and thus the scattering factors for light and heavy atoms are of the same magnitude. Note that unit-cell dimensions as well as space groups sometimes vary between investigations based on XRD and powder neutron diffraction.

Another important point to note is that, most atomic arrangements are determined on powders owing to synthetical difficulties which may have poor crystallinity and show presence of impurity phases (often not detected directly by XRD). For example, the structurally complicated case Mg${}_{6}$Co${}_{2}$D${}_{11}$ [15] has 63 positional parameters, it exhibits structural disorder, and the experimental data have limited resolution when collected with conventional XRD technique. High resolution measurements (say, with synchrotron XRD) are rare. The data are usually analyzed by the Rietveld method. For improved convergence, the number of refinable parameters, in particular those referring to the atomic displacement amplitudes, are reduced.

Owing to the above mentioned reasons, hydride (deuteride) structures may rightfully be said to be less well characterized than other compounds. In this situation, theoretical investigations are also a valuable supplementary tool for the experimentalists by suggesting possible structural arrangements with unit-cell parameters and atomic positions.

### 2.2. Tailor made complex hydrides

Due to high hydrogen content of complex hydrides, interest in these materials has grown considerably. However, the features of such compounds are largely unexplored. A simple complex hydride has the general formula $A_{x}M$H${}_{y}$ where *A* may be an alkali or alkaline-earth metal, and *M* can be almost any of the transition metals from the right-hand side of The Periodic Table. The stoichiometries of such compounds are quite variable, *x* = 1−4 and *y* = 2−9. In addition, some quaternary compounds which include two different *A* elements (counter-ions) are also known (e.g., LiMg(AlD${}_{4}$)${}_{3}$, K${}_{2}$NaAlH${}_{6}$, *etc.*) [16,17]. Moreover, in recent years the mixed alkali-metal and transition metal borohydrides are also known (e.g., LiZn${}_{2}$(BH${}_{4}$)${}_{5}$, NaZn${}_{2}$(BH${}_{4}$)${}_{5}$, *etc.*) [18]. This implies that considerable number of such complexes are possible around 200 examples have been hitherto structurally characterized using mostly the powerful neutron diffraction technique [19].

From the light weight and high hydrogen storage point of view, alkali- and alkaline-earth-metal based hydrides along with Group IV elements (especially B, Al, and Ga) have obtained special attention. Using the simple chemical picture one can identify several hypothetical series of phases, e.g., *A*H (*A* = alkali), *B*H${}_{2}$ (*B* = alkaline-earth element), ABH${}_{3}$, ACH${}_{4}$ (*C* = Group IV element), *B*BH${}_{4}$, $A_{2}BH_{4}$, BCH${}_{5}$, $AB_{2}$H${}_{5}$, $A_{3}B$H${}_{5}$, $A_{3}B$H${}_{6}$, $AB_{3}$H${}_{7}$, $A_{2}B_{3}$H${}_{8}$, $A_{3}B_{3}$H${}_{9}$, $A_{4}B_{3}$H${}_{10}$, *etc.* The *A*H and *B*H${}_{2}$ phases are structurally well characterized and for the ABH${}_{3}$, and ACH${}_{4}$ series most of the compounds are identified. On the other hand, in the remaining series only very few compounds (e.g., in the BCH${}_{5}$ series BaAlH${}_{5}$ [20]; in the $A_{2}B$H${}_{4}$ series Cs${}_{2}$MgH${}_{4}$ Ref. [21]) are experimentally identified and these are often synthesized via high-pressure routes. So far all established complex hydrides have high decomposition temperatures (usually close to the melting point). However, experimental evidences showed that it is possible to reduce the decomposition temperature by controlling the particle size or by addition of suitable “catalytic” material [3,4,6,22]. These findings clearly imply that, it is not unlikely than some other compounds, by some synthesis technique and/or with some additives one may obtain materials with more useful operating temperatures and better kinetics. However, such additive substitution ultimately reduces the total storage capacity (the additives are much heavier) and one may have to go a long way to find such materials. From the point of stored hydrogen amount (regardless of whether the absorption/desorption is reversible), one can tune the H content by varying the chemical composition of the storing system.

## 3. Structural Investigation: A Challenging Task

As mentioned in the previous section, owing to the complexity in structural arrangements and difficulties involved in establishing hydrogen positions by x-ray diffraction methods, structural information are very limited for hydrides [19]. From the high hydrogen content (in wt %) point of view, only limited number of elements and their different combinations can be used for this purpose. Hence, alkali and alkaline-earth metals in combination with group III, IV, and V group elements of The Periodic Table are getting considerable interest. However, within this limitation itself, one can have numerous well-defined series of phases, but, only few members of these series have so far been experimentally explored. Experimentally one can find the crystal structure of the system from XRD, PND, and Raman spectra study. On the other hand no unique method is available for such study on theoretical basis. Several approaches like guess-structure/ICSD (Inorganic crystal structural data base) [23] approach, simulated annealing, genetic algorithm, force-field approach, molecular dynamic study, *etc.* are being used to reach the global minima for a chosen chemical composition. In most of the cases different methods predict different structures. The structures predicted from ICSD mostly agree well with experimental structures (see Ref. [24]). Based on the authors experience, the ICSD approach is more reliable where existing structural information (within similar chemical formula; e.g., $AB_{2}$; *A* and *B* are elements in the Periodic Table) is used as a starting point. In this approach the validity of such prediction depends upon the number of guess structures (more the phases more the reliability). Moreover, most of the binary, ternary, and quaternary (not for all combinations) phases, structural information are available (see Table I) and this approach is more suitable. Even though this theoretical approach has been used for several decades for other classes of materials [25,26] we have pioneered this ICSD-based approach to hydrides and solved the structure of several hydrides, for example KAlH${}_{4}$ [27]. Total energies have been calculated by the projected-augmented plane-wave (PAW) [28,29] implementation of the Vienna *ab initio* simulation package (VASP). [30,31] All these calculations are made with the generalized gradient approximation of the PBE [32,33] exchange correlation functional and the projector augmented wave method. More details about the computational parameters involved in the calculations can be obtained from corresponding original articles.

**Figure 1:**
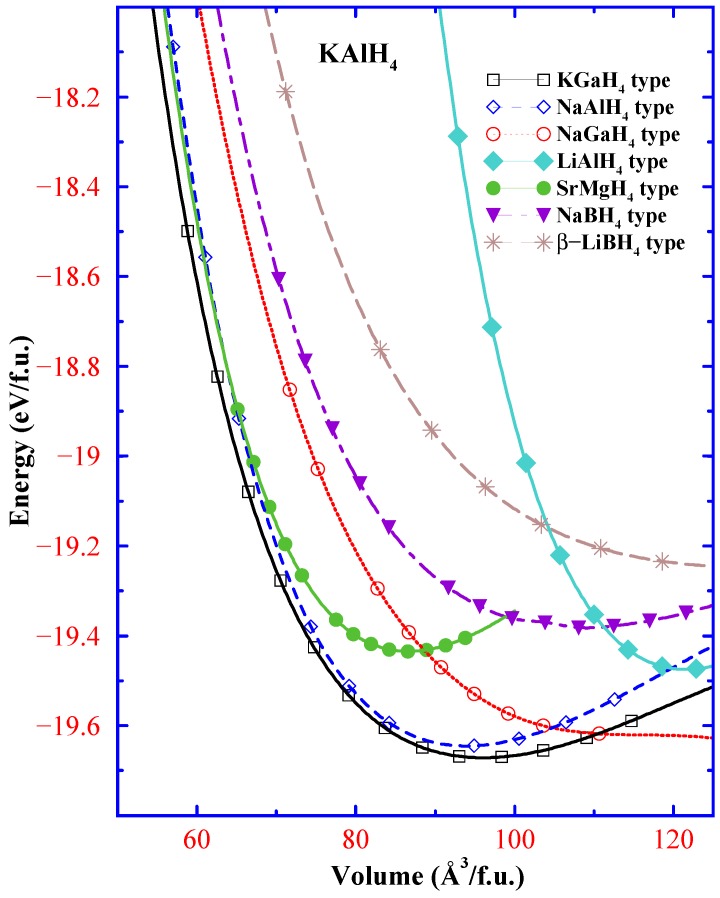
Structural competition between different possible structural arrangements for KAlH${}_{4}$.

Experimental evidence shows that reversible hydrogen absorption/desorption proceeds smoothly in KAlH${}_{4}$ without introduction of a catalyst [7]. For our study seven closely related potential structure types have been considered to find the structure of KAlH${}_{4}$: LiAlH${}_{4}$ (monoclinic; *P2${}_{1}$/c*) [34], *α*-NaAlH${}_{4}$ (tetragonal; $I4_{1}/a$) [35,36], *β*-LiBH${}_{4}$ (hexagonal; $P6_{3}mc$) [37], NaGaH${}_{4}$ (orthorhombic; Cmcm) [38], NaBH${}_{4}$ (cubic, Fm3m) [39], SrMgH${}_{4}$ (orthorhombic; $Cmc2_{1}$) [40], and KGaH${}_{4}$ (orthorhombic; Pnma) [41]. In order to identify the ground-state structure of KAlH${}_{4}$, we have calculated the total energy as a function of cell volume for the seven tested possible structural variants (Figure 1). Among them the orthorhombic KGaH${}_{4}$-type arrangement is seen to have the lowest total energy (see Figure 1) with unit-cell dimensions *a* = 9.009, *b* = 5.767, *c* = 7.399 Å at 0 K and ambient pressure. This prediction has been later verified by Hauback *et al* [42] and the observed unit-cell dimensions *a* = 8.8736, *b* = 5.7253, *c* = 7.2603 Å at 8 K are in very good agreement with the theoretical prediction (see Table 1). This study clearly indicates that one can use DFT as a tool to explore the ground state structure of unknown phases. This approach often becomes very useful to find meta-stable phases [44,45,46,47,48,49,50,51,52,53,54,55,56,57,58] of a chosen compound that may have distinct property than the ground state phase [47,50,59]. We have utilized this approach to investigate crystal structures of a wide variety of hydrides. [e.g., BeH${}_{2}$, MgH${}_{2}$, *A*BeH${}_{3}$, *A*MgH${}_{3}$, *A*BH${}_{4}$, *A*AlH${}_{4}$, *A*GaH${}_{4}$ (*A* = Li, Na, K, Rb, cs), Li${}_{3}$AlH${}_{6}$, Na${}_{3}$AlH${}_{6}$, K${}_{3}$AlH${}_{6}$, *A*(*B*H${}_{4}$)${}_{2}$ (*A* = Mg, Ca,; *B* = B, Al), $M^{\prime }$AlH${}_{4}$ ($M^{\prime }$ = Be, Mg, Ca, Sr, Ba), Ca(AlH${}_{4}$)${}_{2}$; see Refs. [44,45,60,61,62,63,64,65,66,67]. This database searching approach has recently become quite popular and one of the regular tools to find hydride crystal structures [48,68,69,70,71,72,73,74,75].

**Table 1:** Theoretically optimized and experimentally observed ( ${}^{a}$ From XRD measurements [43]; ${}^{b}$ From PND measurements at 8K [42]) structural parameters for KAlH${}_{4}$.

Unit-cell dimensions (Å)	Positional parameters	
	Theory	Experiment
KGaH${}_{4}$ type:	K (4c) : 0.1778, 1/4, 0.1621	K (4c) : 0.1775(7), 1/4, 0.1598(9)
*a* = 9.009 (8.814${}^{a}$; 8.736${}^{b}$)	Al (4c) : 0.5663, 1/4, 0.8184	Al (4c) : 0.5659(6), 1/4, 0.8201(7)
*b* = 5.767 (5.819${}^{a}$; 5.725${}^{b}$)	H1 (4c) : 0.4034, 1/4, 0.9184	D1 (4c) : 0.4063(5), 1/4, 0.9250(4)
*c* = 7.399 (7.331${}^{a}$; 7.260${}^{b}$)	H2 (4c) : 0.7055, 1/4, 0.9623	D2 (4c) : 0.7153(5), 1/4, 0.9611(6)
	H3 (8d) : 0.4194, 0.9810, 0.3127	D3 (8d) : 0.4181(3), 0.9791(4), 0.3137(4)

For reliable structural prediction one should use as much initial structures as possible from the ICSD data base, [23] in order to avoid ending up with wrong structures. For example, for $ABX_{3}$ around 6639 data entries are available in ICSD (see Table 2). Selecting input structures from these 6639 entries for the $ABX_{3}$ composition is itself tedious process and tremendous computations involved for such a large entries. Several compounds/phases having the same structure type and some cases have only small variation in the positional parameters (for certain atoms). These possibilities are omitted because during the full geometry optimization, even though we used different positional parameters, the structures converged mostly to the similar type of structural arrangement. In particular, this $ABX_{3}$ composition has only 30 structure types with unique structural arrangements and hence we have used only these 30 applicable structures. *A*BeH${}_{3}$ and *A*MgH${}_{3}$ (*A* = Li, Na, K, Rb, and Cs) series are part of the $ABX_{3}$ family. Among the *A*BeH${}_{3}$ series, none of the compounds are experimentally identified. Since Be is an extremely toxic element, special precautions are employed in its handling. Therefore, the structure of most of these phases is not yet established experimentally. On the other hand among the *A*MgH${}_{3}$ series all other compounds are experimentally known except LiMgH${}_{3}$. Within the 30 unique structural arrangements, we used only 24 structure models with which we are able to reproduce the structure of the *A*MgH${}_{3}$ series and also predicted the crystal structure of LiMgH${}_{3}$ [76]. On the other hand in the *A*BeH${}_{3}$ family we predicted the structure of the LiBeH${}_{3}$ as orthorhombic (space group Pnma), whereas, a recent theoretical finding by Chao-Hao et al. found the structure as monoclinic (CaSiO${}_{3}$-type structure; space group P2(1)/c) [75] using the same ICSD approach. This deviation can be attributed to the exclusion of ternary halides in our simulation. It should be noted that the structure obtained from evolutionary simulations is much different from the ICSD approach and the former predicts higher-energy structure (for more details see Ref. [75]). This finding clearly implies that the reliability of calculations depends upon the number of input structures considered in the calculations and this method is better than the other currently-available methods.

**Table 2:** Number of Inorganic Crystal Structure Database (ICSD) entries for selected compound types.

ICSD formula	Example	Number of entries	Independent structures
AX	LiH	3710	58
$AX_{2}$	MgH${}_{2}$	3375	98
ABX	KSbZn	391	69
$ABX_{2}$	AgInTe${}_{2}$	17	7
$ABX_{3}$	NaMgH${}_{3}$	6639	30
$ABX_{4}$	LiAlH${}_{4}$	2015	103
$ABX_{5}$	CaAlH${}_{5}$	317	45
$ABX_{6}$	GaBH${}_{6}$	377	32
$AB_{2}X_{4}$	MgCs${}_{2}$H${}_{4}$	4790	131
$AB_{3}X_{4}$	Ag${}_{3}$PO${}_{4}$	226	26
$AB_{3}X_{5}$	MgCs${}_{3}$H${}_{5}$	173	34
$AB_{2}X_{6}$	RuSr${}_{2}$H${}_{6}$	1344	36
$AB_{3}X_{6}$	Li${}_{3}$AlH${}_{6}$	465	43
$AB_{2}X_{7}$	Sr${}_{2}$AlH${}_{7}$	243	34
$AB_{2}X_{8}$	Ca(BH${}_{4}$)${}_{2}$	271	50
$A_{3}B_{4}X_{10}$	Mg${}_{3}$Cs${}_{4}$D${}_{1}0$	6	3
$A_{6}B_{7}X_{26}$	Ba${}_{6}$Mg${}_{7}$D${}_{2}6$	62	12
$ABCX_{5}$	TiTaKO${}_{5}$	127	12
$ABCX_{6}$	LiMgAlH${}_{6}$	158	18
$ABC_{2}X_{6}$	LiAlK${}_{2}$H${}_{6}$	1957	23
$ABC_{3}X_{12}$	CaLi(BH${}_{4}$)${}_{3}$	18	8
$AB_{2}C_{4}X_{16}$	CaLi${}_{2}$(BH${}_{4}$)${}_{4}$	27	9

The ICSD technique offers an efficient way to focus the search by selectively choosing the most likely ground states. However, this method relies heavily on the existence of an extensive database of good trial structures and is incapable of generating new crystal structure types in the absence of information on similar compounds. For the quaternary and multi-component systems one can find a few or no structural inputs at all (see Table 2). Hence, different approach is needed for such cases. The main drawback of other methods is, the optimization mostly leads to a local minima instead of global minima. These methods are more suitable for energetic studies where the structure need not to be the completely correct one (the error bar within 10 KJ/mole). The cluster expansion technique allows one to efficiently search the configuration space of alloys, but it is currently limited to lattice-based systems [77]. Recently, genetic algorithms have emerged as promising methods for finding new ground state crystal structures in systems that either have diffraction data or can be described by classical potentials [78], but the computational expense of first-principles DFT calculations combined with the genetic algorithm for large systems currently limits their applicability to unit cells with a few tens of atoms [79,80]. Methods based on first-principles variable cell shape molecular dynamics [81] can be used to accurately explore structural transformation paths, but they require the user to supply a good starting structure and become difficult to apply when such data is not available. Monte Carlo-based techniques have been used for the structure determination of a wide range of systems, from finite clusters [82] and organic molecules, [83] to crystalline solids [84,85,86]. Due to their versatility and generality, these methods are capable of predicting not only the ground states, but also entropically stabilized phases [82]. However, with a few notable exceptions [78] Monte Carlo methods have been seldom used systematically in conjunction with accurate first-principles DFT energetics in the search for new materials. Such applications usually require extensive and time-consuming fitting of interatomic potentials. [85] search methods have been explored with some success toward solving the structure of Lennard-Jones clusters and small molecules [87]. Recently, Majzoub and Ozolin$\overset{˘}{s}$ have tried to solve the structure of complex bulk crystals with multiple ionic species [70]. Even though several methods are formulated till date they all have limitation to the type of bonding, number of atoms, *etc.*

From the lattice dynamic study one can evaluate the dynamical stability of the predicted crystal structure. Linear response, or density functional perturbation theory, is one of the most popular methods of *ab initio* calculation of lattice dynamics however, the applicability of the method extends beyond the study of vibrational properties. The basic theory of phonons, or lattice vibrations, in crystals is well understood and has been described in detail in several textbooks which is beyond the aim of this review. In a stable crystal all the phonon frequencies must be positive. An optimization of the crystal structure under constrains of the space group symmetry elements may lead to an atomic configuration, which does not yet correspond to a global energy minimum. In this case some phonon frequencies may appear as negative values (soft modes). In hydrides, it is often found that one or more structures are dynamically stable (e.g., see Ref. [24,69] ) for a single compound. Validation of such structures using phonon study is not discussed in this review that will be addressed elsewhere.

### Magnesium borohydride Mg(BH${}_{4}$)${}_{2}$: A challenging case

The predicted structures from first principle methods mostly fit well with the experimental findings. However, this is not true always; the computation models are strictly valid for defect-free solid at 0 K. In reality the samples can be impure and defective. Hence some times the predicted structure from the above-mentioned ICSD or other approaches may not fit with the experimental findings. Magnesium borohydride, Mg(BH${}_{4}$)${}_{2}$ is one such typical example. Mg(BH${}_{4}$)${}_{2}$, appears to be a promising material for hydrogen-storage applications. Upon heating, it decomposes to release 14.9 wt % hydrogen (theoretical) according to reaction below [88].

(1) $$\mathrm{Mg}{\left({\mathrm{BH}}_{4}\right)}_{2}\to \mathrm{Mg}+2B+4H_{2}$$

Although synthesis of this compound was first reported more than 50 years ago, the structure of unsolvated Mg(BH${}_{4}$)${}_{2}$ remains elusive. Furthermore, the literature data on the synthesis and properties are contradictory, likely because of the presence of different solvates and the difficulty in removing the solvent molecules without decomposition. Plešek and HeřmáLnek isolated unsolvated magnesium borohydride using the reaction of MgH${}_{2}$ with diborane [89]. Konoplev and Bakulina reported the synthesis of unsolvated Mg(BH${}_{4}$)${}_{2}$ via an exchange reaction, and published a qualitative reflection list from x-ray powder diffraction (XRD) data of two crystal modifications [88]. However, the poor quality of the diffraction data precluded determination of the crystal structure. Empirical calculation of the enthalpy of decomposition of Mg(BH${}_{4}$)${}_{2}$ [90,91] gave a very attractive value of ca. 40 kJ mol${}^{-1}$ H${}_{2}$, which would suggest that the hydrogen release should be reversible at moderate temperatures. Interest in this system has stimulated a number of theoretical attempts [64,68,92,93,94,95]. Unfortunately, all studies predicted different crystal structures for Mg(BH${}_{4}$)${}_{2}$ from the experimentally determined structure.

**Figure 2:**
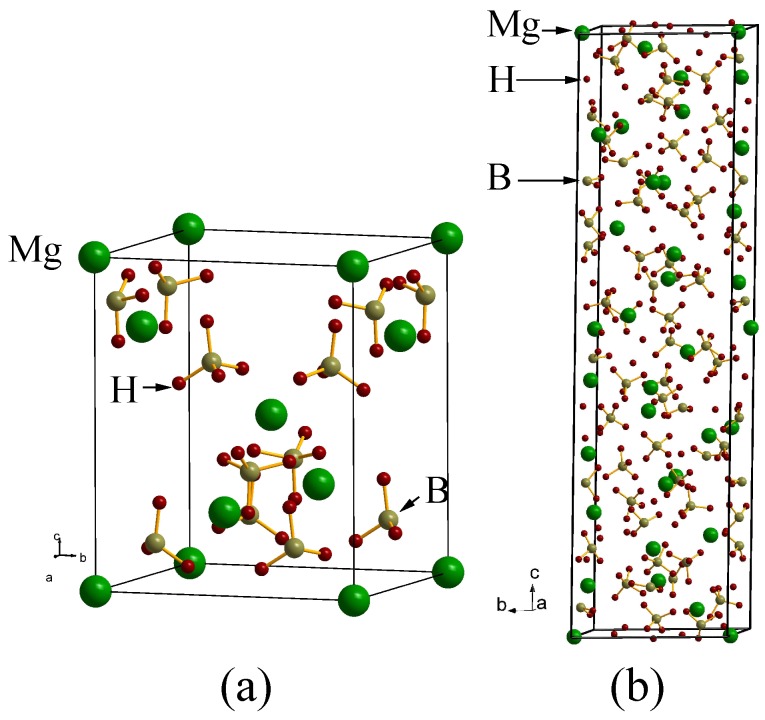
Crystal structure of Mg(BH${}_{4}$)${}_{2}$: (a) from theoretically obtained low energy structure (Tetragonal I4m2), (b) experimentally identified low temperature structure (Hexagonal, $P6_{1}$).

From the experimental side, the structure of Mg(BH${}_{4}$)${}_{2}$ is critically dependent on the experimental conditions (especially final heat treatment). Different procedures can yield either or *α* or *β* or both of the phases. Keeping the sample below 453K results in the formation of the low-temperature (*α*, Hexagonal, $P6_{1}$) phase, while the high-temperature (*β*, Orthorhombic, Fddd) phase dominates if the temperature exceeds 508K. Subsequent cooling does not cause the transformation of the *β* phase back to the *α* phase. Intermediate temperatures usually give a mixture of both *α* and *β* phases [96]. From the theoretical side the total-energy density functional theory (DFT) calculations of Mg(BH${}_{4}$)${}_{2}$ in 28 relatively simple structure types suggested that, the most-likely ion arrangement is that corresponding to the monoclinic Cd(AlCl${}_{4}$)${}_{2}$ type, albeit with a somewhat higher (orthorhombic, Pmc2${}_{1}$) symmetry [64]. On the other hand, similar DFT calculations by other authors predicted different modifications, one having a Mg(AlH${}_{4}$)${}_{2}$-like ion arrangement and trigonal symmetry, and the other having a novel ion arrangement and monoclinic symmetry [68]. Clearly, all these structure models differ substantially. The only common features for the reported structures is the low dimensionality (sheet-like for the orthorhombic and trigonal models, and chain-like for the monoclinic model) and their simplicity (one symmetry independent cation site and up to two symmetry-independent anion sites). Recent theoretical findings by Ozolins et al. [95] predict the structure as tetragonal I4m2, using structure prototype electrostatic ground state search strategy. This I4m2 structure is 5.4 kJ/mol lower in energy than the low temperature $P6_{1}22$ structure found in Refs. [96,97,98] and this structure is relatively simpler (see Figure 2a) than the experimentally identified low temperature $P6_{1}22$ structure. Very recently, Zhou *et al.* [99] have predicted two other structural models, one tetragonal structure with space group I4122 and another orthorhombic structure with space group F222, having lower in energy than so far found experimental as well as theoretical structures. Unlike other borohydrides, Mg(BH${}_{4}$)${}_{2}$ has a remarkably complex structure (see Figure 2b). The relative sizes of the ions allow for an 8-fold coordination of Mg${}^{2+}$ by H atoms, supplied in pairs by 4(BH${}_{4}$)${}^{-}$ ions, resulting in a coordination shell that has the shape of a dodecahedron. Each of these is linked to 4 neighboring dodecahedra by (BH${}_{4}$)${}^{-}$ bridges generating a tetrahedral network. The shape of the dodecahedra and the torsions introduced by the bridges do not allow for a simple, high symmetry network and lead to a complex structure.

From the literature data on the synthesis and properties are sometime contradictory, likely because of the presence of different solvates and the difficulty in removing the solvent molecules without decomposition. According to the experimental study, the LT $P6_{1}22$ phase contains an unoccupied void, accounting for 6.4% of space in the structure. It is large enough (37 Å${}^{3}$) to accommodate a small molecule, such as H${}_{2}$O. The high-temperature phase is less dense by ∼3% but contains no unoccupied voids (Ref. [98]). The deviation between the theory and the experimental structures are hypothesized by the authors in [95] that interactions with solvent molecules may be responsible for the nucleation and growth of the $P6_{1}22$ structure in solution-grown crystals. One has to remember that the theoretically predicted structures are strictly valid at T = 0 K and most of the cases theory can reproduce the low temperature phase that has lower in energy. Several much simpler hypothetical structures for Mg(BH${}_{4}$)${}_{2}$ [similarly for Ca(BH${}_{4}$)${}_{2}$] have been proposed that have DFT total energies close to the low-temperature ground-state structure. In this respect, Mg(BH${}_{4}$)${}_{2}$ may be similar to silica, which has a large number of different crystal structures having about the same energy. It is still not exactly clear why the very complicated 330 atom structure is preferred over the alternative structures, although they have almost similar Mg-B and B-H bond lengths and nearly ideal BH${}_{4}$ tetrahedra seem to be important factors. More research is needed to fully understand the polymorphism of Mg(BH${}_{4}$)${}_{2}$ and also, different synthesis routes may be needed to get the solvent free Mg(BH${}_{4}$)${}_{2}$.

## 4. Search for Potential Metastable Phases

AlH${}_{3}$ is an unique binary hydride having at least six crystalline phases with different physical properties and at the same time stores up to 10.1 wt % of hydrogen [100]. Its gravimetric hydrogen density is two times higher than liquid hydrogen and much higher than that of most of the known metal hydrides. Moreover, elemental Al is a commonly available and recyclable which could be an acceptable component for the future sustainable energy society. Thus, AlH${}_{3}$ is considered as a possible hydrogen storage material [101].

AlH${}_{3}$ is one of the unique compounds that has several polymorphs and the possible reason for such existence has been explained by theory. The crystal structure of *α*-AlH${}_{3}$ has been well studied [102] in the literature and less attention has been focused on the other polymorphs. Recent theoretical study using this ICSD approach by Ke *et al.* [48] found two new phases of AlH${}_{3}$ which are energetically more favorable than the stable *α*-modification. Followed by this study Brinks *et al.* [103,104] and Yartys *et al.* [105] experimentally solved the structure of $\alpha^{\prime }$, *β*-, and *γ*-AlH${}_{3}$ phases. The structural aspects of irradiated AlH${}_{3}$ in comparison with the various phases are also investigated in Ref. [106]. Similarly the electronic structure [48,107] and thermodynamic stability [108] of *α*-AlH${}_{3}$ are also well studied. The high pressure study by Graetz *et al.* [109] observed no pressure induced structural transition in AlH${}_{3}$ up to at least 7 GPa, which is consistent with earlier high pressure studies [110,111]. The pressure dependence on the electronic structure is also discussed in Ref. [109]. A recent high pressure study by Goncharenko *et al.* shows that, application of pressure on *α*-modification transforms it into two different modifications hp1 and cubic hp2 phase at ca 60 and 100 GPa, respectively (the structure of the hp1-phase has not yet been solved experimentally) [59]. As the high pressure diffraction studies are unable to identify the exact positions of hydrogen atoms owing to its very low scattering cross section along with the diamond anvil cell involve in the high pressure study, theoretical knowledge about its stability at high pressure is very important. In our recent work [47] we have verified the presence of such pressure-induced structural phase transition in AlH${}_{3}$. In addition we have solved the structure of hp1-phase and found that these predicted phases are dynamically stable at high pressures [112]. In this study we have used 58 independent structural arrangements (for more details see Ref. [47]) to find the high pressure phases as well as stability of the AlH${}_{3}$ at ambient conditions.

**Figure 3:**
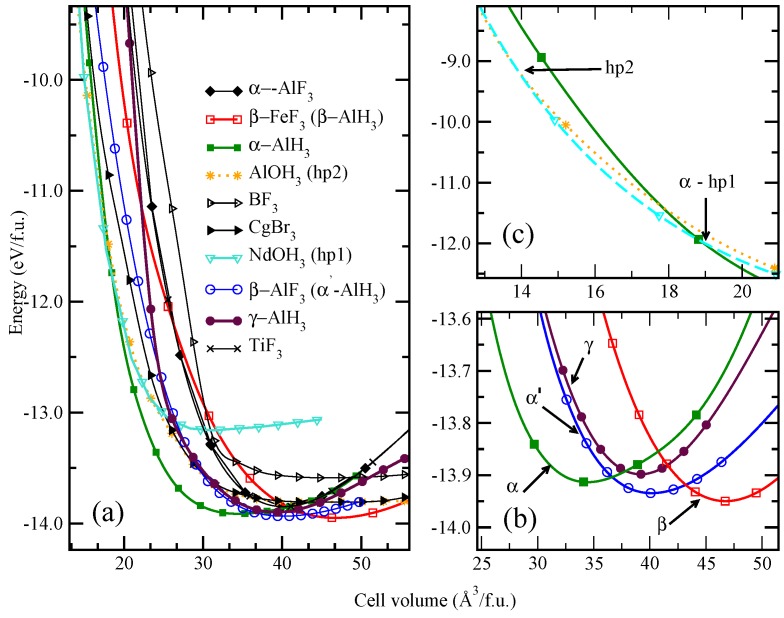
(a) Calculated volume versus total energy curves for AlH${}_{3}$. Magnified versions of the corresponding transition points are shown on (b) and (c) at right-hand side of the figure.

Among the considered structures (for more details see [47]), the *β*-FeF${}_{3}$-type atomic arrangement is found to have the lowest total energy (referred hereafter *β*-AlH${}_{3}$). The calculated positional and lattice parameters are found to be in good agreement (see Table 1 in [47]) with recent experimental findings by Brinks *et al.* [104] and theoretical work by Ke *et al.* [48] The next energetically favorable phase is orthorhombic *β*-AlF${}_{3}$-type (space group Cmcm; $\alpha^{\prime }$-AlH${}_{3}$) atomic arrangement and the involved energy difference between this phase with *β*-AlH${}_{3}$ at the equilibrium volume is only ca. 32.6 meV/f.u. (see Figure 3). The calculated structural parameters are found to be in good agreement (see Table I in Ref. [47]) with the recent experimental finding [103].

Yartys *et al.* [105] solved the structure of *γ* modification and found that it has an orthorhombic structure with the space group Pnnm. But this *γ* modification is found to be 30 meV/f.u. higher in energy than α′-AlH${}_{3}$ at equilibrium volume. As the *γ* phase is higher in energy than the other polymorphs in the whole volume range, it may be experimentally stabilized by temperature. Similar to the α′ modification *γ* modification also has open pores. Hence, both modifications have almost similar equilibrium volumes (Figure 3b). The next energetically favorable structure is *α*-AlH${}_{3}$. The involved energy difference between the *α*- and *β*-AlH${}_{3}$ phase is found to be only 32.6 meV/f.u. It is interesting to note that the involved energy difference between the *α*-, $\alpha^{\prime }$-,*β*-, and *γ*-AlH${}_{3}$ is very small, hence one structure can easily be transformed into another by application of temperature or pressure. However, the experimental findings show that, depending upon the synthesis route/conditions one can stabilize different polymorphs of AlH${}_{3}$ [102,103,104,105]. One should be aware that it is not easy to define the clear boundary about energy difference between the structures from the DFT. If the energy difference between two structures is within 50 meV, it is much easier to switch over from one structure to another. However, in KAlH${}_{4}$ the tetragonal *α*-NaAlH${}_{4}$-type phase is energetically very closer (energy difference is only 25 meV) to the KGaH${}_{4}$-type ground-state phase. However, the presence of such meta-stable phase is hitherto not yet identified experimentally. This may indicate that not only the energetics but also the barrier height to transform from one structure to another structure is important to stabilize the metastable phases.

As mentioned above, according to theory *β*-AlH${}_{3}$ is the ground state structure and application of pressure on *β*-AlH${}_{3}$ transforms it into $\alpha^{\prime }$- modification at 2.4 GPa (see Figure 3). Further application of pressure on this $\alpha^{\prime }$- modification transforms into *α* modification at 4.3 GPa. This *α* modification is experimentally found to be the most stable structure at ambient condition and one can store it for several years without losing H${}_{2}$ [102]. Further application of pressure shows that the *α*- modification transforms into NdOH${}_{3}$ derived modification [see Figure 3b; hp1-AlH${}_{3}$ (P63/*m*) modification] around 64 GPa. Recently it is reported (Ref. [59]) that the hp1 phase can be either monoclinic or trigonal. On the other hand a recent theoretical investigation by Pickard *et al.* [51] shows that the hp1-phase is orthorhombic (Pnma). But our finding shows that the hp1 phase rather has a hexagonal (P63/*m*) structure with the lowest energy among all these phases and the orthorhombic (Pnma) structure suggested in [51] is energetically closer to the presently predicted *P*63/*m* structure. The increase of pressure above 104 GPa brings up a new cubic polymorph (hp2-AlH${}_{3}$ modification) which is consistent with recent experimental findings and this phase has metallic character [59]. We have also made lattice dynamical calculations for all the high pressure phases of AlH${}_{3}$ and found no negative phonon frequencies indicating that all these phases are expected to be dynamically stable. So, one can conclude that the presently predicted hp1 (*P*63/*m*) phase should be the intermediate-pressure-phase, observed experimentally from high pressure measurements [59]. Recent theoretical findings demonstrate that the metallic nature of the electronic structure (for the hp2 phase) entails a more favorable hydrogen removal energy which is lowered by 75% compared to the insulating *α* phase [50]. It might be possible that the cubic meta-stable phase could be prepared and stabilized experimentally at ambient pressure by off-board quenching. The above example is a clear indication for theory as a powerful tool to explore possible meta-stable phases that might have peculiar property than the equilibrium phase.

## 5. Stabilizing Meta-Stable Phases by Substitution

As mentioned in the previous section it might be possible to find the meta-stable phases from theoretical simulation. The next question is how can one stabilize such novel high pressure phases by experimental technique? For example, combined theoretical and experimental investigations show that the cotunnite-type structure of TiO${}_{2}$ (synthesized at pressure above 60 GPa and high temperatures) has been shown [113,114] to exhibit an extremely high bulk modulus (431 GPa) and hardness (38 GPa). Subsequent processing involving rapid decompression could lead to the existence of this phase in a metastable state at ambient pressure. Similarly, some meta-stable hydride phases may have better kinetics that could solve the hydrogen storage problem. Hence, searching such meta-stable phases becomes one of the prime interests.

MgH${}_{2}$ is one of the potential candidates for hydrogen storage application that can store up to 7.6 wt % of hydrogen [115,116,117]. The major drawback of this material is the rate at which hydrogen absorbs and desorbs due to the fact that the diffusion of hydrogen atoms through the hydride is slow. The hydrogen molecules do not readily dissociate at the surface of Mg to generate the hydrogen atoms that diffuse into the metal. Transition metals can catalyse this bond breaking/formation event at the surface, but not main group elements [116,118,119,120]. The hydrogen atoms in MgH${}_{2}$ bind too strongly with the Mg atoms, *i.e.,* the enthalpy of formation of the hydride is too large (−76.2 ± 9.2 kJ mol${}^{-1}$), [121] so that the hydride needs to be heated to very high temperature, around 350 ${}^{\circ }$C, in order to release hydrogen gas at high enough pressure (over 1 atm) [115,116,117]. In order to use MgH${}_{2}$ as energy carrier in mobile applications, one has to find the possible ways to decrease the hydrogen desorption temperature. Numerous studies have been focused on improving the problematic sorption kinetics, including mechanical ball milling [9,10,122] and chemical alloying [11,12]. However, it is found [118] that these methods can only improve absorption and not desorption kinetics, possibly because even the smallest particle sizes (20 nm)obtainable by these methods still primarily display bulk desorption characteristics.

**Figure 4:**
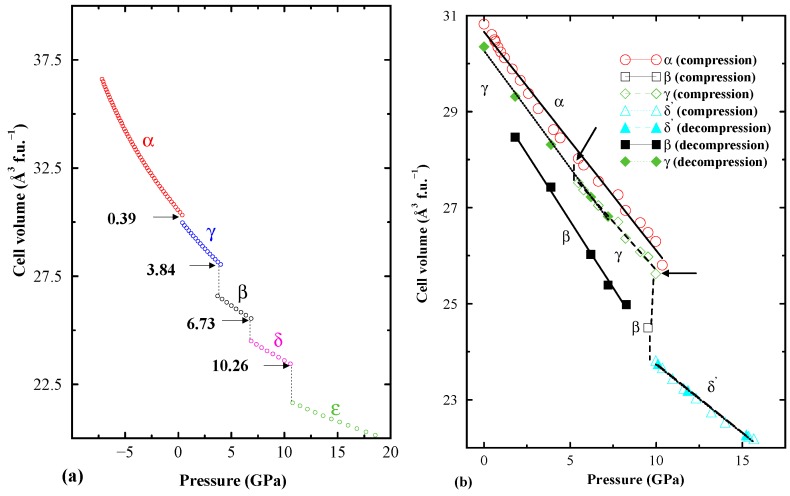
Theoretically calculated (left panel) and experimentally observed (right panel) pressure *vs.* volume relation for MgH${}_{2}$. Pressure stability regions for the different modifications are indicated.

In [45] many pressure-induced transitions in MgH${}_{2}$ have been predicted by the authors [45], and subsequent experimental finding confirmed such pressure-induced structural transitions [123]. *α*-MgH${}_{2}$ crystallizes with TiO${}_{2}$-r-type (r = rutile) structure at ambient pressure and low temperature [124,125]. At higher temperatures and pressures tetragonal *α*-MgH${}_{2}$ transforms into orthorhombic *γ*-MgH${}_{2}$. We have calculated the energy as a function of volume for 11 closely related structures (for more details see Ref. [45]). At 0.387 GPa (Figure 4a), *α*-MgH${}_{2}$ transforms into *γ*-MgH${}_{2}$ and the total energy of the two modifications is nearly the same at the equilibrium volume, it is only natural that these phases coexist in a certain volume range [126]. On application of pressure *γ*- to *β*-MgH${}_{2}$ transition occurs at 3.84 GPa. Formation of such high pressure *β* modification occurs experimentally [123,124]. In the pressure range from 6.7 to 10.2 GPa the structural arrangements of the *β*, *δ*, and $\delta^{\prime }$ modifications lie within a narrow energy range of some 10 meV, a further transformation to *ϵ*-MgH${}_{2}$ is predicted at 10.26 GPa, but this is not verified experimentally. This closeness in energy suggests that the relative appearance of these modifications will be quite sensitive to, and easily affected by, external factors like temperature and remanent lattice stresses, as well as by kinetics. In this connection it should be reminded that the theoretical simulation relates to a defect-free pure phase at 0 K, whereas the high-pressure diffraction experiments were performed at room temperature on a sample burdened with likely defects and impurities.

The theoretically obtained and the experimentally measured pressure vs volume relations are displayed in Figure 4. At ambient pressure (1 bar) and room temperature MgH${}_{2}$ stabilizes in the TiO${}_{2}$-rutile-type structure with space group *P*4${}_{2}$/mnm [124,125]. From the experimental findings; during compression *γ*-MgH${}_{2}$ starts to form at 5.5 GPa and coexists along with *α*-MgH${}_{2}$ up to some 9.5 GPa. In a narrow pressure window between 9.35 and 10.36 GPa, the *β*-MgH${}_{2}$ polymorph (modified CaF${}_{2}$-type structure) exists in a three-phase mixture with the *α* and *γ* modifications. This sequence of the experimentally established high-pressure polymorphs generally agrees with the theoretical predictions, although the observed transition pressures deviate somewhat from the calculated values. The latter findings may reflect that neither entropy nor temperature effects are taken into account in the theoretical simulation, and furthermore, the experimental sample is not 100% pure. In addition, nucleation of new phases at a first-order transition may have slow kinetics. At pressure above 10 GPa the experimentally established *α*, *γ*, *β* phase-mixture transforms into an AuSn${}_{2}$-type phase (this polymorph being hereafter denoted $\delta^{\prime }$). This phase is structurally quite different from the theoretically predicted orthorhombic *δ* phase (space group Pbc2${}_{1}$). However, a closer look at Figure 1 in Ref. [45] shows that the energy difference between these two modifications is indeed very small, less than 1 meV. Hence, the discrepancy between theory and experiment may be explained as metastability of the *δ*${}^{\prime }$ phase, e.g., invoked by the particular pressure sequence used experimentally.

Considerable hysteresis is observed (See Figure 4b) in the phase changes upon pressure release. The *δ*${}^{\prime }$ phase transforms into the *β* polymorph at 9.85 GPa under decreasing pressure. This phase remains stable and constitutes the dominating phase at 6.23 GPa. The *α*, *γ* mixture finally converts into a single-phase product of *γ*-MgH${}_{2}$ at 1.79 GPa. Hence, *γ*-MgH${}_{2}$ remains as the final product after the completed decompression cycle.

It should be highlighted that from 6.23 to 9.85 GPa pressure regime the *β* polymorph becomes stabilized. In general, it is widely believed that the cubic modifications may have lower decomposition and better kinetics than the other modification. In this point of view the high pressure *β* polymorph gets special attention. If one can stabilize this high pressure phase, it might have better hydrogen storage properties than the other polymorphs like *α* and *γ*. Recently Kyoi et al synthesis the Mg${}_{7}$TiH${}_{x}$ phase at high pressure (8 GPa) and high temperature (873 K) and this phase crystalizes in cubic form (Fm3m) [127]. The hydrogen desorption temperature of this compound is about 130 K lower than for the hydrogen desorption of MgH${}_{2}$. Mg${}_{7}$TiH${}_{x}$ structure is a super structure of the high pressure *β*- modification. It should be noted that not only Ti, but, V, Zr, Nb, Hf and Ta can also stabilize such high pressure cubic phase as a ambient phase [127,128,129,130,131,132]. In this aspect theory is a powerful tool to explore such metastable phases which can later be stabilized by either rapid quenching or substitution. However, experimentally it is more difficult to scan such possible meta-stable phases in large categories of samples. Therefore conducting theoretical simulation can save man power, money, and environment.

## 6. Conclusions

First-principles density-functional calculations, previously a tool only for the theoretical physicist, can now be broadly applied to several areas of materials research, many of which have direct relevance to industry. Further evidence of the value of this approach can be found in our recent efforts aimed to solve the structures of new hydrogen storage materials. At present, none of the material exhibits the combination of high hydrogen densities, low desorption temperatures, fast kinetics, and low cost needed for automotive applications. New hydrides with desirable properties must be developed. In this review we have demonstrated how the state-of-the-art density functional calculations can be used to reproduce/predict the crystal structure of the known as well as unknown phases from the guess-structure/ICSD approach. As an example, the ground-state crystal structure of KAlH${}_{4}$ has been identified from the structural optimisation of a number of structures using force as well as stress minimizations. Some times the metastable phase of a chosen compound might have peculiar properties than the ambient phase. Finding such phases from the experimental studies is a challenging task where conducting such theoretical simulations is advantageous. For example, at ambient pressure AlH${}_{3}$ stabilizes in the *β*-FeF${}_{3}$-type structure. From the simulation we have found that various modifications of AlH${}_{3}$ can be obtained by applying different amounts of pressure similar to obtaining AlH${}_{3}$ by different preparatory conditions. The high pressure meta-stable phase has novel metallic character. It might also be possible to stabilize such meta-stable phase by substitution which is demonstrated in the case of MgH${}_{2}$. The high-pressure polymorphs of *β*-MgH${}_{2}$ modification is stabilized by Ti/V/Zr/Nb/Hf substitution that has less decomposition temperature than the *α* or *γ* MgH${}_{2}$. The ICSD technique offers an efficient way to focus this search by selectively choosing the likeliest ground states. However, these methods rely heavily on the existence of an extensive database of good trial structures and are incapable of generating new crystal structure types in the absence of information on similar compounds. More research in this area is highly desirable, especially in cases where (for example) the database of candidate structure prototypes is limited.
